# Single-Substance SSRI Intoxication: A Clinical and Outcome Profile Presentation in a Poisoning Referal Center

**DOI:** 10.1155/emmi/4727543

**Published:** 2025-05-03

**Authors:** Rokhsareh Meamar, Zahra Rabiei, Awat Feizi, Melika Namvar, Nastaran Eizadi-Mood

**Affiliations:** ^1^Department of Clinical Toxicology, School of Medicine, Isfahan Clinical Toxicology Research Center, Khorshid Hospital, Isfahan University of Medical Sciences, Isfahan, Iran; ^2^Isfahan University of Medical Sciences, Isfahan, Iran; ^3^Department of Epidemiology and Biostatistics, School of Public Health, Isfahan University of Medical Sciences, Isfahan, Iran; ^4^Isfahan Clinical Toxicology Research Center, Khorshid Hospital, Isfahan University of Medical Sciences, Isfahan, Iran

**Keywords:** cardiac complications, intoxication, QT interval prolongation, serotonin syndrome, SSRI overdose

## Abstract

**Background:** Due to the increasing concern about selective serotonin reuptake inhibitors (SSRIs) poisoning, specifically the risk of serotonin syndrome (SS), and the QT-prolonging effects of certain SSRIs, we evaluated the clinical presentations and outcomes of patients who overdosed on single SSRIs.

**Methods:** We carried out a cross-sectional study at a Poisoning Emergency referal center in Isfahan, Iran, involving 101 patients who had taken a single SSRI drug and were hospitalized between January 2021 and January 2024. Information on demographics, toxicological features, clinical symptoms, electrocardiogram (ECG) findings, and outcomes was gathered.

**Results:** The average age of the patients was 26.98 ± 10.57 years. Females outnumbered males (male to female ratio was 1:3.8). Sertraline was the most frequently ingested SSRI (43.6%), followed by fluoxetine (18.8%). Gastrointestinal symptoms (nausea and vomiting) were the most common clinical signs (*n* = 30, 29.7%). Six patients (5.9%) were diagnosed with SS. Only one patient experienced a brief, self-limiting seizure after consuming 4 g of sertraline. ECG showed QT interval prolongation (QT prolongation) in 32 patients (31.6%). One patient developed a first-degree AV block after taking 600 mg of citalopram. There was no significant difference in QT prolongation or SS based on the type of SSRI used. All patients survived without complications.

**Conclusion:** This study indicates that overdosing on a single SSRI typically results in mild to moderate clinical manifestations. Cardiac issues, such as QT prolongation, were relatively common among our patients.

## 1. Introduction

Selective serotonin reuptake inhibitors (SSRIs), commonly prescribed antidepressants, were introduced in the 1980s for the treatment of depression. They quickly became a key component in the pharmacological management of major depressive episodes and anxiety disorders [1].

Major depression is a common disorder and a significant health problem. It is ranked as the 13^th^ leading cause of disability and mortality worldwide in 2019, with a lifetime prevalence of 12% [2]. In 2022, the United States' Poisoning Center reported that SSRIs were involved in 32,520 cases of single-substance toxic exposure with a 2.45% fatality rate in all exposures associated with SSRIs [3]. According to a systematic review and meta-analysis, SSRIs are the 14th leading cause of poisoning in Iran, with an incidence rate of 1.3% [4].

SSRI overdose as a sole agent rarely causes a fatal outcome [5]. SSRIs possess a wide therapeutic window, as doses over 30 times the therapeutic range may result in no symptoms or mild symptoms, and doses over 50–75 times can cause vomiting, mild central nervous system (CNS) symptoms, and tremors [6]. Since 2011, a controversial discussion about the potential effects of SSRIs on QT interval has emerged. This discussion arose due to postmarketing reports of QT lengthening and torsades de pointes, prompting the FDA to issue a warning to the public and healthcare professionals regarding QT interval prolongation (QT prolongation) with the use of citalopram [7].

Patients who experience adverse effects from SSRIs may exhibit a range of symptoms known as serotonin syndrome (SS), a potentially life-threatening condition that occurs following the use of serotonergic drugs. The prevalence of SS has been increasing in recent years due to the rising prescription rates of serotonergic psychiatric medications [8]. Patients with SS may experience altered mental status, autonomic hyperactivity, and neuromuscular abnormalities [9]. According to Mikkelsen et al., 10%–14% of patients who have ingested a single-substance SSRI have developed SS, with most cases displaying mild to moderate symptoms. The most commonly implicated SSRIs in cases of SS were sertraline, paroxetine, and fluvoxamine [10].

Seizures and severe CNS depression are uncommon outcomes of SSRI overdoses [10]. According to one study analyzing 469 SSRI overdoses, seizures have been observed in 1%–2% of patients [10].

In light of the growing concern regarding SSRI poisoning, particularly the potential for life-threatening SS, and the debate surrounding the QT-prolonging effects of certain SSRIs, further investigation into the clinical outcomes of SSRI overdose is warranted [11]. This study aims to address this gap in knowledge by examining the medical outcomes and toxic manifestations observed in patients admitted to a referral hospital in the center of Iran following SSRI ingestion.

## 2. Method

**Figure 1:**
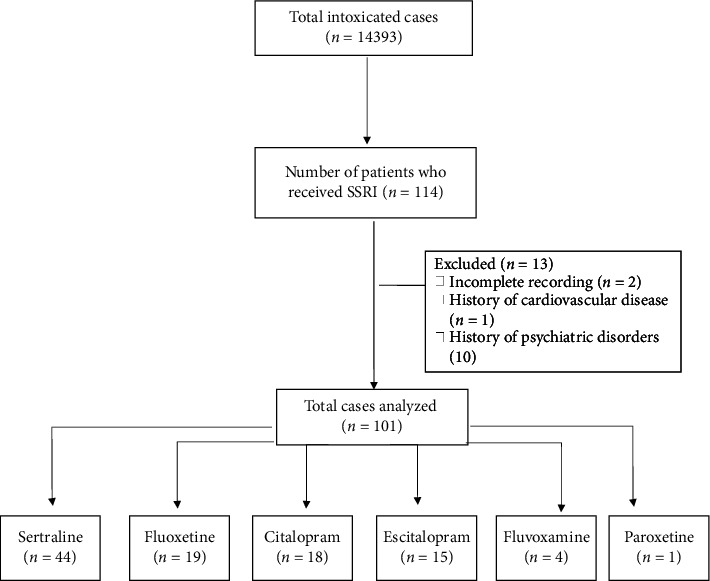
Flowchart.

This cross-sectional study was conducted at the Poisoning Emergency Department of Khorshid Hospital, affiliated with Isfahan University of Medical Sciences, in Isfahan, Iran. This center serves as a primary referral center for poisoning cases in Isfahan, the central part of Iran. The hospital's archive was utilized to access the available medical documents in order to collect patients' information. All patients who were admitted for a single SSRI overdose between January 2021 and January 2024 were included. Patients with coingestion of other drugs, as well as two or more SSRIs, and those with a previous history of cardiovascular disease and psychiatric disorders were excluded (Figure 1).

### 2.1. Data Collection

Using a data collection form, all data concerning demographic information (including age, sex, marital status, and level of education) and toxicological characteristics (including type of SSRI ingested, amount of drug taken in number and dose, coingestion of other drugs, time span from ingestion to hospital admission, and duration of hospitalization), as well as clinical manifestations including blood pressure, respiratory rate, pulse rate, and body temperature at the time of admission and 6 h later, were collected.

In addition, laboratory parameters, including renal function tests (creatinine [mg/dL] and BUN [mg/dL]), venous blood gas (VBG), creatinine phosphokinase (CPK), lactate dehydrogenase (LDH) (U/L), partial thrombin time/prothrombin time (PTT/PT), and INR measured at admission, as well as electrocardiography (ECG) findings, treatment protocol (activated charcoal and 5-HT2 receptor antagonist [cyproheptadin])) were gathered in the data collection form.

### 2.2. Definition

SSRI poisoning is defined as consumption of doses greater than the recommended maximal daily dose: fluoxetine (> 60 mg), fluvoxamine (> 200 mg), paroxetine (> 60 mg), sertraline (> 200 mg), and citalopram (> 60 mg) [10]. The diagnosis of SSRI poisoning was determined by the reported history of the patient or their companions.

Standard 12-lead electrocardiography (ECG) is conducted for all patients upon admission to assess cardiac outcomes, including QT prolongation and arrhythmia. The mean QT interval value from all 12 leads of the ECG is used for QT interval examination. The QT interval is defined from the beginning of the Q wave to the end of the T wave. Using the Bazett correction formula, the QTc interval is calculated as QTc = QT/RR. The RR interval represents the time between two consecutive R waves. QT interval changes (> 0.43 s in men and > 0.45 in women) are considered as QT prolongation [12].

Arrhythmia is considered as any rhythm that is not a normal sinus rhythm with normal atrioventricular conduction [13].

SS is diagnosed based on the Hunter Serotonin Toxicity Criteria: neuromuscular excitation, autonomic stimulation, and changes in mental state [14]. Fever is also defined as a body temperature greater than 38°C.

### 2.3. Objectives

Our primary objective was to assess initial cardiac manifestation among patients with SSRI poisoning. Our secondary objective was to assess all clinical manifestations and outcomes among these groups of patients.

### 2.4. Ethical Consideration

Written informed consent was obtained from all patients. All guidelines as per the Declaration of Helsinki and good clinical practice guidelines were followed. The study protocol was approved by the Ethical Research Committee of Isfahan University of Medical Sciences (with the code: IR.MUI.MED.REC.1402.025).

### 2.5. Statistical Analysis

Data were analyzed using SPSS software, Version 21 (IBM Corp., released 2012, IBM SPSS Statistics for Windows, Version 21.0, Armonk, New York: IBM Corp.). Results were presented as frequency (percent) for qualitative variables and mean ± standard deviation (SD) for quantitative variables. The normality of continuous variables was assessed using the Kolmogorov–Smirnov test and Q-Q plot. Pearson's chi-squared/Fisher's exact tests were utilized to evaluate the association of categorical variables, while independent samples *t*-test or Mann–Whitney nonparametric test were used to compare normally and non-normally distributed continuous variables, respectively, between patients who experienced and did not experience clinical outcomes. A *p* value < 0.05 was considered statistically significant.

## 3. Result

During the study period, 101 single-substance SSRI–intoxicated patients were included in our study. The mean (SD) age of the study population was 26.98 ± 10.57 years. Females outnumbered males (male : female ratio was 1 : 3.8). Sixty patients (59.4%) were married, and 76.3% (*n* = 97) of patients had attained at least a secondary middle school education or higher.

The median (minimum, maximum) time interval between self-poisoning and presentation to the toxicology unit was 3 (0.5, 31.0) hours. The length of stay in the hospital for the majority of patients (45.5%) was 0–12 h. About one out of four patients were asymptomatic (24.8%). Gastrointestinal manifestations including nausea and vomiting were the most common clinical symptoms (*n* = 30, 29.7%) followed by fatigue (*n* = 16, 15.8%) and vertigo (*n* = 10, 9.9%). Eighty (79.2%) patients experienced no vomiting. Sertraline was the most commonly ingested SSRI (43.6%), followed by fluoxetine (18.8%). Activated charcoal was administered to 100 patients (99%) and gastric lavage was performed in 77 patients (76.2%).

Only one 14-year-old girl experienced a seizure during hospitalization, which was short and self-limiting, after taking 4000 mg of sertraline. She did not exhibit any signs of SS or cardiac complications.

Table 1 compares between patients with and without SS and cardiac outcomes including QT prolongation and arrhythmia based on demographic and toxicological characteristics.

### 3.1. SS

Six patients (5.9%) developed SS, all of whom were women. The majority of them consumed sertraline (*n* = 4, 66.7%) followed by citalopram (*n* = 1, 16.7%) and escitalopram (*n* = 1, 16.7%). No significant differences were observed in terms of age, sex, level of education, time interval between consumption and hospitalization, length of stay, type of SSRI consumed, chief complaint, and number of vomiting between patients with SS and those without (*p* > 0.05).

Signs, symptoms and clinical findings of patients on admission and 6 h later are presented in Table 2. Results showed a significant difference in presentation of agitation, tremor, fever, HTN, and myoclonus between patients with SS and those without SS (all *p* < 0.05). Among the SS group, 66.7% had tremor (*n* = 4), 50% had fever (*n* = 3), 33.3% had myoclonus (*n* = 2), and 66.7% had HTN (*n* = 4) upon admission. Patients with SS had significantly higher mean SBP (141.67 ± 10.41) and DBP (86.0 ± 9.38) compared to those without SS at the time of admission. Moreover, subjects with SS exhibited significantly higher mean (SD) heart rate (119 ± 23.75) and temperature (37.51 ± 0.58) upon admission, compared to patients who did not develop SS (*p* < 0.05). No significant differences were observed in the mean (SD) of SBP, DBP, and temperature 6 h after admission and initiation of medical treatment. However, the mean (SD) of HR remained higher among patients with SS after 6 h between the two groups (*p* < 0.05). In addition, there was a significant decrease in the mean difference of SBP and temperature (*p* < 0.05). Except for O_2_ saturation 6h after admission“, O_2_ saturation and respiratory rate showed no significant differences at admission, 6 hours later, or in the mean difference between these two time points (*P* > 0.05). Diarrhea, mydriasis, and diaphoresis were not observed in any patients.

All 6 patients were treated with a 5-HT2a antagonist (cyproheptadine).

### 3.2. Cardiac Manifestation

By evaluating the cardiovascular indices obtained from the ECG on admission, it was found that 32 (31.6%) patients developed QT prolongation and 4 (3.9%) patients had arrhythmia including 3 patients with sinus tachycardia and 1 patient with Grade 1 AV block (Table 1). A 23-year-old female patient, experienced Grade 1 AV block after being poisoned with 600 mg of citalopram. A 19-year-old female patient who took 1200 mg of sertraline had inverted T waves in her precordial leads, while troponin levels remained negative.

The mean (SD) corrected QT interval in the QT-prolonged group was 483.84 ± 26.17 ms, while in patients with no QT prolongation was 432.29 ± 19.85 (*p* < 0.001). Sertraline consumption was determined to be higher in patients with QT prolongation compared to other SSRI drugs (*n* = 14, 43.8%), followed by escitalopram (*n* = 6, 18.8%), and fluoxetine (*n* = 5, 15.6%). Similarly, sertraline was the most common SSRI in patients with arrhythmia. Both QT prolongation and arrhythmia mainly involved women (75.0% and 100%, respectively), but it was not statistically significant (Table 1). In addition, in the group with QT prolongation, a significant difference was observed in the number of vomiting episodes compared to those without QT prolongation (*p* value = 0.016).

**Figure 2:**
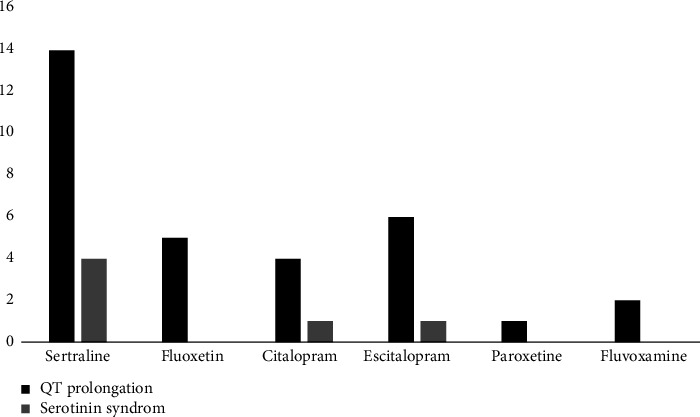
Comparing SSRI drug types based on QT prolongation and serotonin syndrome.

A bar chart comparing SSRI types and associated complications, including QT prolongation and SS, is presented in Figure 2.

In Table 2, patients with arrhythmia showed a significant difference based on HTN when compared to patients without HTN (*p*=0.007). Agitation and fever were higher in patients with QT prolongation (*p*=0.03). In addition, a significant increase in HR was observed at admission and 6 h later in the group with arrhythmia compared to patients without arrhythmia (*p* < 0.001). There was no statistically significant difference in either QT prolongation or arrhythmia with respect to age, level of education, the time interval between consumption and hospitalization, the type of SSRI drug used, and chief complaint (*p* > 0.05) when compared to patients without QT interval prolongation or arrhythmia.

A nonsignificant difference was detected in patients with QT prolongation compared to those without QT prolongation according to NGT, lavage, and BNZ treatment (Table 3).

There were no significant differences in terms of laboratory findings including creatinine, BUN, VBG, CPK, LDH, PTT/PT, and INR between patients with SS, QT prolongation and arrhythmia when compared with patients without these complications. Only one patient had CPK > 1000 (2990) after being poisoned with 750 mg of sertraline.

All the patients were discharged from the hospital after suitable treatment and complete recovery. There was no mortality in any of the patients.

## 4. Discussion

Comparative studies on poisoning with antidepressants have indicated lower mortality rates and less severe sequelae with SSRIs [15]. Nevertheless, severe toxicity could still occur. The present study provided toxico-clinical manifestations of patients with SSRI toxicity in the Poisoning Emergency Department of Khorshid Hospital. This study helps identify the spectrum of effects associated with SSRI poisoning.

In line with other studies [10], our results revealed that the majority of SSRIs are safe in overdose as no cases of death were observed. However, there are some reports of death following massive SSRI overdose [16, 17].

Consistent with previous research [10], the majority of our patients overdosed on sertraline.

In a study of 21 patients with SSRI poisoning, only one patient had a QTc interval greater than 500 milliseconds (520 milliseconds), which was associated with sertraline consumption [18]. Whereas, we reported that sertraline consumption was determined to be higher in patients with QT prolongation compared to other SSRI drugs. A case report suggests that sertraline may have the potential to cause QT prolongation [19]. QTc interval normalized after sertraline withdrawal, indicating that sertraline contributed to the increase in QTc interval. In a randomized, 3-way crossover, double-blind study, a positive signal for QTc prolongation was observed for sertraline at a steady-state dose of 400 mg/day dose [20]. Alternatively, sertraline could potentially worsen QTc prolongation if used concurrently with other drugs known to increase the risk of QTc prolongation. Therefore, it is possible that concurrent drug use may have contributed to this toxicity. In addition, it should be noted that all data are based solely on self-reports from patients and do not include serum drug concentrations. However, the magnitude of QT prolongation was not specified.

However, an FDA meta-analysis of randomized controlled trials found that sertraline had a lower risk of “suicidality” compared to other antidepressants [21].

Our results mirrored previous reports [10] regarding the low incidence of seizure in SSRI poisoning, occurring in only one patient with sertraline overdose in our study. Rare reports exist of brief self-limiting seizures on sertraline [10, 22, 23]. Seizures with sertraline have also been reported in patients receiving therapeutic doses, particularly in those with prolonged treatment duration, higher treatment doses, and situations that make patients prone to seizures [23–25].

Furthermore, the majority of QT prolongation cases (43.8%) and SS (66.7%) occurred in patients who took sertraline, but no statistical difference was seen. A review of 469 SSRI overdoses reported that sertraline accounted for 20% of SS [10]. SSRIs are commonly implicated medicines associated with SS, tending to have mild manifestations after isolated ingestion [6]. While SS is rare, it can be life-threatening [26].

Consistent with our findings that SS occurred in 5.9% of the patients. The incidence of SS after an overdose of a single-agent SSRI is reported to be about 14%–16% [27]. According to a review of The Toxicology Investigators Consortium (ToxIC) registry, sertraline, citalopram, fluoxetine, escitalopram, and paroxetine were among the top 10 agents associated with SS [28].

Similar to a previous study [27], our results showed agitation, tremor, myoclonus, HTN, and fever and had a statistically significant association with SS diagnosis. A retrospective study of 1010 patients with SS reported that clonus and hyperreflexia were the most common clinical manifestations present in 60% of patients [28].

In our study, sertraline accounted for the majority of QT prolongations followed by escitalopram, fluoxetine and citalopram. SSRIs were initially thought to not have significant cardiotoxicity compared with TCAs [29]. In recent years, this newer class of antidepressants has been suggested to have potential cardiovascular complications [30]. In a meta-analysis of 16 prospective controlled studies, it was observed that SSRIs exhibited a dose-dependent increase in QTc interval when compared to the administration of a placebo [31]. SSRIs, especially citalopram, can antagonize myocyte potassium channels leading to QT prolongation, which may trigger torsades de pointes and fatal reentrant tachycardias [32–34]. In a logistic regression analysis of 57 patients with citalopram poisoning, it was reported that citalopram was 5 times more likely to cause QT > 440 msec than sertraline [10]. In a prospective, cross-sectional population-based study, sertraline (*n* = 42) was associated with an increase in QTcF by a mean of 1.7 milliseconds (90% CI: −3.4–6.9) [35]. A single-center, randomized, double-blind, placebo- and moxifloxacin-controlled thorough QT study reported a positive signal for QTc interval prolongation with sertraline at the steady-state of 400 mg/day dose [36]. In a cross-sectional study, 41 out of 103 patients with sertraline overdose had QTc > 440 msec with 6 patients experiencing QTc > 500 ms [10].

Nevertheless, in our study, there was no significant statistical difference observed in terms of the kind of SSRI and QT prolongation. The limited sample size in our study may account for this.

We also observed a case of first-degree AV block following ingestion of 600 mg citalopram. To our knowledge, research regarding the association of citalopram and AV blockage is limited to a few case reports [37]. Some other cardiac abnormalities observed with citalopram intoxication include left bundle branch block [10], transient right bundle branch block [38], sinus tachycardia [39], sinus bradycardia [40], and supraventricular tachycardia [41].

Our study had several limitations. First, we relied on a single EKG for cardiac analysis. Second, drug concentrations were not measured for patients in this study due to the fact that they were not part of the routine diagnostic protocol at our institution, so some cases of overdose are over- or underestimated. It is important to note that in the past history, we only reported the psychiatric history of patients without asking about the specific types of drugs they were taking, such as SSRIs, tricyclics, or antipsychotics. Due to this limitation, we were unable to confirm whether patients were regularly taking SSRIs before overdosing or if it was an acute exposure. However, this study is based on a single-center hospital, so the representativeness of findings may be limited.

## 5. Conclusion

In conclusion, this study investigated the clinical outcomes and toxic manifestations observed in patients admitted to a Poisoning referal center following an SSRI overdose. SS was identified in 5.9% of patients. QT prolongation was observed in 31.6% of patients and arrhythmia was identified in 3.9% of patients. This study highlights the importance of considering the potential for cardiac complications following SSRI overdose; however, the long-term cardiac complications of SSRI overdose remain unclear. Future studies should assess whether transient QT prolongation observed in acute overdose cases increases the risk of persistent cardiac abnormalities.

### 5.1. Implications for Research

More research is needed to determine the comparison of SS and ECG findings in patients with and without a past history of taking SSRI as an antidepressant medication.

### 5.2. Implications for Clinician

Although mortality is rare in SSRI poisoning, it may be practical to educate general physicians and psychiatrists about the consequences of exposure to this toxic agent.

## Figures and Tables

**Table 1:** Comparison of demographic and toxicologic and chief complaint based on serotonin syndrome, arrhythmia, and QT interval prolongation in patients with SSRI intoxication.

		Total *N* = 101	Serotonin syndrome *N* = 6	No serotonin syndrome *N* = 95	*p* value	Arrhythmia *N* = 4	No arrhythmia *N* = 97	*p* value	QT prolongation *N* = 32	No QT prolongation *N* = 69	*p* value
Age		26.98 ± 10.57	23.50 ± 12.11	27.20 ± 10.50	0.41	25.00 ± 3.56	27.06 ± 10.76	0.70	26.78 ± 10.65	27.07 ± 10.62	0.90

Sex	Female	80 (79.2%)	6 (100%)	74 (92.5%)	0.24	4 (100.0%)	76 (78.3%)	0.30	24 (75.0%)	56 (81.2%)	0.48
	Male	21 (20.8%)	0 (0.0%)	21 (22.1%)		0 (0.0%)	21 (21.6%)		8 (25.0%)	13 (18.8%)	

Education level	Illiterate	4 (4.0%)	0 (0.0%)	4 (4.2%)	0.89	0 (0.0%)	4 (4.1%)	0.76	1 (3.1%)	3 (4.3%)	0.82
	Primary school	20 (19.8%)	2 (33.3%)	18 (18.9%)		1 (25.0%)	19 (19.6%)		8 (25.0%)	12 (17.4%)	
	Secondary school	49 (48.5%)	3 (50.0%)	46 (48.4%)		1 (25.0%)	48 (49.5%)		13 (40.6%)	36 (52.2%)	
	B.Sc	25 (24.8%)	1 (16.7%)	24 (25.3%)		2 (50.0%)	23 (23.7%)		9 (28.1%)	16 (23.2%)	
	M.Sc	3 (3.0%)	0 (0.0%)	3 (3.2%)		0 (0.0%)	3 (3.1%)		1 (3.1%)	2 (2.9%)	

Time interval			4.13 ± 2.98	5.06 ± 5.50	0.68	5.63 ± 3.94	4.98 ± 5.44	0.81	4.76 ± 6.36	5.12 ± 4.90	0.76

Length of stay	0–12 h	46 (45.5%)	2 (33.3%)	44 (46.3%)	0.82	0 (0.0%)	46 (47.4%)	0.17	13 (40.6%)	33 (47.8%)	0.29
	12–24 h	40 (39.6%)	3 (50.0%)	37 (38.9%)		3 (75.0%)	37 (38.1%)		16 (50.0%)	24 (34.8%)	
	> 24 h	15 (14.9%)	1 (16.7%)	14 (14.7%)		1 (25.0%)	14 (14.4%)		3 (9.4%)	12 (17.4%)	

Kind of SSRI	Sertraline	44 (43.6%)	4 (66.7%)	40 (42.1%)	0.8	3 (75.0%)	41 (42.3%)	0.75	14 (43.8%)	30 (43.5%)	0.51
	Fluoxetine	19 (18.8%)	0 (0.0%)	19 (20.0%)		0 (0.0%)	19 (19.6%)		5 (15.6%)	14 (20.3%)	
	Citalopram	18 (17.8%)	1 (16.7%)	17 (17.9%)		1 (25.0%)	17 (17.5%)		4 (12.5%)	14 (20.3%)	
	Escitalopram	15 (14.9%)	1 (16.7%)	14 (14.7%)		0 (0.0%)	15 (15.5%)		6 (18.8%)	9 (13.0%)	
	Fluvoxamine	4 (4.0%)	0 (0.0%)	4 (4.0%)		0 (0.0%)	1 (1.0%)		1 (3.1%)	0 (0.0%)	
	Paroxetine	1 (1.0%)	0 (0.0%)	1 (1.1%)		0 (0.0%)	4 (4.1%)		2 (6.3%)	2 (2.9%)	

Chief complaint	Asymptomatic	25 (24.8%)	1 (16.7%)	24 (25.3%)	0.95	0 (0.0%)	25 (25.8%)	0.58	12 (37.5%)	13 (18.8%)	0.27
	Nausea/vomiting	30 (29.7%)	3 (50.0%)	27 (28.4%)		1 (25.0%)	29 (29.9%)		8 (25.0%)	22 (31.9%)	
	Confusion	1 (1.0%)	0 (0.0%)	1 (1.1%)		0 (0.0%)	1 (1.0%)		1 (3.1%)	0 (0.0%)	
	Dizziness	8 (7.9%)	0 (0.0%)	8 (8.4%)		1 (25.0%)	7 (7.2%)		2 (6.3%)	6 (8.7%)	
	Vertigo	10 (9.9%)	1 (16.7%)	9 (9.5%)		0 (0.0%)	10 (10.3%)		3 (9.4%)	7 (10.1%)	
	Agitation	4 (4.0%)	0 (0.0%)	4 (4.2%)		0 (0.0%)	4 (4.1%)		2 (6.3%)	2 (2.0%)	
	Dyspnea	5 (5.0%)	0 (0.0%)	5 (5.3%)		0 (0.0%)	5 (5.2%)		0 (0.0%)	5 (7.2%)	
	Fatigue	16 (15.8%)	1 (16.7%)	15 (15.8%)		2 (50.0%)	14 (14.4%)		4 (12.5%)	12 (17.4%)	
	Palpitation	2 (2.0%)	0 (0.0%)	2 (2.1%)		0 (0.0%)	2 (2.1%)		0 (0.0%)	2 (2.9%)	

The number of vomiting	0	80 (79.2%)	4 (66.7%)	76 (80.0%)	0.37	3 (75.0%)	77 (79.4%)	0.81	27 (84.4%)	53 (76.8%)	0.016
	1	12 (11.9%)	2 (33.3%)	10 (10.5%)		1 (25.0%)	11 (11.3%)		1 (3.1%)	11 (15.9%)	
	2	6 (5.9%)	0 (0.0%)	6 (6.3%)		0 (0.0%)	6 (6.2%)		1 (3.1%)	5 (7.2%)	
	3	3 (3.0%)	0 (0.0%)	3 (3.2%)		0 (0.0%)	3 (3.1%)		3 (9.4%)	0 (0.0%)	

Abbreviation: SSRIs = selective serotonin reuptake inhibitors.

**Table 2:** Clinical findings at admission and 6 h later in patients with serotonin syndrome, arrhythmia, and QT prolongation.

		Total *N* = 101	Serotonin syndrome *N* = 6	No serotonin syndrome *N* = 95	*p* value	Arrhythmia *N* = 4	No arrhythmia *N* = 97	*p* value	QT prolongation *N* = 32	No QT prolongation *N* = 69	*p* value
Agitation	Yes	3 (3.0%)	2 (33.3%)	1 (1.1%)	0.009	1 (33.3%)	3 (3.1%)	0.12	3 (9.4%)	0 (0.0%)	0.030
	No	98 (97.0%)	4 (66.7%)	94 (98.9%)		2 (66.7%)	95 (96.9%)		29 (90.6%)	69 (100.0%)	

Seizure	Yes	1 (1.0%)	0 (0.0%)	1 (1.1%)	0.94	0 (0.0%)	1 (1.0%)	0.96	0 (0.0%)	1 (1.4%)	0.68
	No	100 (99.0%)	6 (100.0%)	94 (98.9%)		4 (100.0%)	96 (99.0%)		32 (100.0%)	68 (98.6%)	

Tremor	Yes	4 (4.0%)	4 (66.7%)	0 (0.0%)	< 0.001	1 (25.0%)	3 (3.1%)	0.15	2 (6.3%)	2 (2.9%)	0.38
	No	97 (96.0%)	2 (33.3%)	95 (100.0%)		3 (75.0%)	94 (96.9%)		30 (93.8%)	67 (97.1%)	

Fever	Yes	3 (3.0%)	3 (50.0%)	0 (0.0%)	< 0.001	0 (0.0%)	3 (3.1%)	0.89	3 (9.4%)	0 (0.0%)	0.03
	No	98 (97.0%)	3 (50.0%)	95 (100.0%)		4 (100.0%)	94 (96.9%)		29 (90.6%)	69 (100.0%)	

HTN	Yes	4 (4.0%)	4 (66.7%)	0 (0.0%)	< 0.001	2 (50.0%)	2 (2.1%)	0.007	2 (6.3%)	2 (2.9%)	0.38
	No	97 (96.0%)	2 (33.3%)	95 (100.0%)		2 (50.0%)	95 (97.9%)		30 (93.8%)	67 (97.1%)	

Myoclonus	Yes	2 (2.0%)	2 (33.3%)	0 (0.0%)	0.003	1 (25.0%)	1 (1.0%)	0.078	2 (6.3%)	0 (0.0%)	0.10
	No	99 (98.0%)	4 (66.7%)	95 (100.0%)		3 (75.0%)	96 (99.0%)		30 (93.8%)	69 (100.0%)	

SBP		119.76 ± 12.59	141.67 ± 10.41	118.38 ± 11.42	< 0.001	131.25 ± 21.75	119.29 ± 12.03	0.062	119.03 ± 12.78	120.10 ± 12.59	0.69

SBP 6 h		116.07 ± 9.66	123.00 ± 9.38	115.63 ± 9.56	0.070	122.75 ± 11.87	115.79 ± 9.53	0.16	115.61 ± 9.42	116.28 ± 9.83	0.75

DBP		76.61 ± 9.02	86.0 ± 9.38	74.96 ± 8.64	0.003	83.50 ± 13.38	75.29 ± 8.75	0.074	74.13 ± 9.87	76.30 ± 8.59	0.26

DBP 6 h		74.22 ± 8.74	79.83 ± 6.21	73.86 ± 8.78	0.10	74.50 ± 11.68	74.21 ± 8.67	0.95	73.90 ± 8.97	74.36 ± 8.70	0.81

Different SBPs		−3.64 ± 10.97	−18.67 ± 8.43	−2.68 ± 10.43	< 0.001	−8.50 ± 15.59	−3.44 ± 10.80	0.37	−3.23 ± 11.16	−3.83 ± 10.96	0.80

Different DBPs		−1.44 ± 9.37	−6.17 ± 8.33	−1.14 ± 9.40	0.20	−9.00 ± 7.62	−1.13 ± 9.34	0.10	−0.32 ± 8.83	−1.94 ± 9.63	0.43

RR		17.04±±2.38	18.17 ± 0.41	16.97 ± 2.43	0.23	17.00 ± 2.00	17.04 ± 2.40	0.97	17.59 ± 2.34	16.78 ± 2.63	0.11

RR 6 h		17.27 ± 1.83	17.83 ± 2.14	17.23 ± 1.81	0.44	17.75 ± 2.06	17.25 ± 1.82	0.59	17.39 ± 2.044	17.22 ± 1.73	0.67

Different RRs		0.26 ± 2.20	−0.33 ± 2.34	2.20 ± (−5.00)	0.50	0.75 ± 2.99	0.24 ± 2.18	0.65	−0.13 ± 2.29	0.43 ± 2.16	0.24

HR		92.16 ± 19.31	119.00 ± 23.75	90.46 ± 17.83	< 0.001	126.25 ± 17.02	90.75 ± 18.14	< 0.001	94.19 ± 22.42	91.22 ± 17.79	0.48

HR 6 h		87.09 ± 14.59	104.83 ± 14.18	85.96 ± 13.94	0.002	112.75 ± 12.15	86.02 ± 13.73	< 0.001	87.48 ± 17.75	86.91 ± 13.07	0.86

Different HRs		−5.14 ± 15.07	−14.17 ± 19.07	14.71 ± (−49.00)	0.13	−13.50 ± 11.39	−4.79 ± 15.15	0.26	−7.00 ± 20.28	−4.30 ± 12.11	0.41

O_2_ saturation		95.90 ± 2.26	96.00 ± 1.55	95.89 ± 2.30	0.91	96.75 ± 1.71	95.87 ± 2.28	0.45	95.97 ± 1.98	95.87 ± 2.39	0.84

O_2_ saturation 6 h		96.19 ± 1.31	97.50 ± 1.05	96.11 ± 1.28	0.011	97.25 ± 1.26	96.15 ± 1.30	0.098	96.13 ± 1.20	96.22 ± 1.36	0.76

Different O_2_ saturations		0.28 ± 2.09	1.50 ± 1.52	2.10 ± (−4.00)	0.14	0.50 ± 2.08	0.27 ± 2.10	0.83	0.13 ± 1.94	0.35 ± 2.16	0.63

Temperature 0		36.95 ± 0.29	37.51 ± 0.58	36.92 ± 0.22	< 0.001	37.00 ± 0.00	36.95 ± 0.29	0.73	36.97 ± 0.42	36.94 ± 0.19	0.63

Temperature 6 h		36.91 ± 0.21	37.03 ± 0.20	36.90 ± 0.21	0.13	36.93 ± 0.10	36.91 ± 0.21	0.85	36.96 ± 0.22	36.88 ± 0.20	0.096

Different temperatures		−0.05 ± 0.33	−0.48 ± 0.49	−0.02 ± 0.30	0.001	−0.75 ± 0.10	−0.05 ± 0.33	0.88	−0.03 ± 0.42	−0.06 ± 0.28	0.67

*Note:* HTN = hypertension; 6 h = 6 hours after admission.

Abbreviations: DBP = diastolic blood pressure; HR = heart rate; RR = respiratory rate; SBP = systolic blood pressure.

**Table 3:** Comparison of treatment protocol in patients based on serotonin syndrome, arrhythmia, and QT prolongation.

		Total *N* = 101	Serotonin syndrome *N* = 6	No serotonin syndrome *N* = 95	*p* value	Arrhythmia *N* = 4	No arrhythmia *N* = 97	*p* value	QT prolongation *N* = 32	No QT prolongation *N* = 69	*p* value
NGT + lavage	Yes	77 (76.2%)	6 (100.0%)	71 (74.7%)	0.19	4 (100.0%)	73 (75.3%)	0.33	28 (87.5%)	49 (71.0%)	0.07
	No	24 (23.8%)	0 (0.0%)	24 (25.3%)		0 (0.0%)	24 (24.7%)		4 (12.5%)	20 (29.0%)	

Charcoal	Yes	100 (99.0%)	6 (100.0%)	94 (98.9%)	0.94	4 (100.0%)	96 (99.0%)	0.96	32 (100.0%)	68 (98.6%)	0.68
	No	1 (1.0%)	0 (0.0%)	1 (1.1%)		0 (0.0%)	1 (1.0%)		0 (0.0%)	1 (1.4%)	

BZD	Yes	10 (9.9%)	0 (0.0%)	10 (10.5%)	0.53	1 (25.0%)	9 (9.3%)	0.35	2 (6.3%)	8 (93.8%)	0.33
	No	91 (90.1%)	6 (100.0%)	85 (89.5%)		3 (75.0%)	88 (90.7%)		30 (11.6%)	61 (88.4%)	

*Note:* BZD = benzodiazepine; NGT = nasogastric tube.

## Data Availability

The data that support the findings of this study are available on request from the corresponding author.
